# Wheat resistance to Fusarium head blight and breeding strategies

**DOI:** 10.1007/s44297-025-00048-1

**Published:** 2025-04-08

**Authors:** Haigang Ma, Yongjiang Liu, Suhong Zhang, Jianfeng Sha, Yawen Sun, Zhiruo Hu, Linjun Gong, Yi Dai, Yujiao Gao, Yonggang Wang, Hongxiang Ma

**Affiliations:** 1 Jiangsu Key Laboratory of Crop Genomics and Molecular Breeding/Zhongshan Biological Breeding Laboratory/Key Laboratory of Plant Functional Genomics of the Ministry of Education, Agricultural College of Yangzhou University, Yangzhou, China; 2 Jiangsu Co-Innovation Center for Modern Production Technology of Grain Crops, Jiangsu Key Laboratory of Crop Genetics and Physiology, Yangzhou University, Yangzhou, China; 3 Yangzhou Modern Seed Innovation Institute, Gaoyou, China

**Keywords:** Wheat, Fusarium head blight, Resistance, Mechanism, Breeding, Variety

## Abstract

Fusarium head blight (FHB) in wheat is one of the most damaging diseases affecting global wheat production. Over the past few decades, significant advancements have been made in mitigating the impact of this disease through the development of resistant wheat varieties. However, the FHB epidemic has been increasing due to changes in climate and crop rotation. Improving breeding efficiency is urgently needed. Cloning disease resistance genes and elucidating their molecular mechanisms will accelerate the breeding of FHB-resistant wheat varieties. This review outlines the five types of FHB resistance in wheat, provides definitions and potential mechanisms for each type, and reviews the cloned resistance genes and the resistance mechanisms they mediate. Additionally, this review discusses the progress in breeding FHB-resistant wheat via resistance genes and proposes strategies for different resistance requirements in breeding, with the goal of increasing the efficiency of FHB resistance breeding in wheat.

## Introduction

Fusarium head blight (FHB), caused by the *Fusarium graminearum* species complex (FGSC), is a pervasive and destructive disease in wheat worldwide. The FGSC includes at least 16 phylogenetically distinct species, with the predominant species responsible for FHB varying across different countries [1, 2]. This complex of filamentous fungi typically invades wheat spikes during anthesis, where it produces mycotoxins that reduce grain yield and quality [3]. Notably, the FGSC exploits a wide range of cereal hosts, including wheat, barley, rye, maize, and rice, and is therefore notoriously infamous [4, 5].

Since its outbreak in Anhui and Jiangsu Provinces in China in 1936, wheat FHB has gradually become a common and severe disease in major Chinese wheat-growing areas, especially in the middle and lower reaches of the Yangtze River [6]. From 2000 to 2018, the disease affected more than 4 million hectares annually in China, accounting for ~ 23% of the total wheat-growing area, with average annual yield losses of more than 3.41 million tons [3, 6]. In the United States, wheat FHB was first reported in 1891 in Indiana, and it began to prevail across the country in the early 1990s [7]. From 1993 to 2014, the cumulative economic loss caused by FHB reached over 17 billion dollars in the USA [6]. Additionally, other wheat-growing regions, such as South America, Europe, and Africa, also suffer significant economic losses caused by FHB [8].

The first documentation of wheat FHB epidemics dates back to 1884 in England [7]. Since then, the global fight against the disease has persisted, with significant progress achieved in both understanding its pathogenesis and developing control strategies. As a pivotal strategy for FHB management, genetic resistance derived from wheat and its relatives has been successfully integrated into modern breeding programs, leading to the release of many FHB-resistant wheat varieties worldwide [7, 9]. Advanced scientific technologies have facilitated the cloning of key resistance genes from these resistance germplasms, and the mechanisms they mediate are being elucidated, further aiding FHB resistance breeding.

## Wheat resistance to FHB

### Types of disease resistance

Resistance to FHB in wheat is acknowledged to be a polygenic quantitative trait, implying that it is influenced by multiple genes, with each contributing small effects to the overall resistance phenotype. Despite extensive research, no immune sources or immune genes that confer complete resistance to FHB have been identified, highlighting the complexity and polygenic nature of the trait. FHB resistance in wheat can be classified into five distinct types, each representing different aspects of the resistance response (Table 1) [10–12].

**Table 1:** Five types of resistance in wheat to Fusarium head blight

Resistance type	Definition	Connotations	Resistance mechanisms	Assessment	References
Type I	prevents the initial infection of the fungus	a) impedes the colonization and germination of fungal spores; b) impedes the development and spreading of hyphae on the surface of spikelets; c) prevents hyphal entry into wheat cells	a) improves wheat ability to recognize pathogens; b) triggers the outburst of extracellular hydrogen peroxide; c) induces stomatal closure; d) strengthens cell wall thickness	a) conidiospore spraying or grain-spawn inoculation; b) calculating the percentage of diseased spikelets in a spike, or the percentage of diseased spikes in a plot	[8–15]
Type II	inhibits the spread of symptoms within the spike	a) impedes pathogens spread from the infected spikelet into the rachis; b) prevents pathogens spread from the rachis to other spikelets	inhibits or abolishes the functions of the pathogenic factors (effector proteins, DON, fusaoctaxin A, etc.)	a) single floret inoculation method; b) calculating the percentage of symptomatic spikelets	[8–10, 14, 17, 18, 20, 22–24]
Type III	kernel resistance to mycotoxin accumulation	a) inhibits the secretion of pathogenic toxins in the grains; b) transforms or degrades the toxins in the grains	a) partially overlap with those of Type II resistance; b) the most remains unclear	harvest all seeds after inoculation, and measure mycotoxins after drying	[8–10, 28]
Type IV	resistance to kernel infection	resistance level expressed in wheat grains	similar to Type I, Type II or Type III	calculating the proportion of Fusarium-damaged kernels in the total harvested kernels	[8–10, 30, 31]
Type V	tolerance to yield loss	plant's ability to withstand disease at varying levels	inhibition of fungal growth	comparing the relative reduction in yield between diseased and healthy plants of the same variety	[8–10, 32]

**Type I** resistance prevents the initial infection of the fungus, serving as the first line of defense against FHB. Resistance is associated with mechanisms that impede the colonization and germination of fungal spores, as well as the development and spread of hyphae on the surface of host spikelets [13, 14]. It may also involve preventing hyphal entry into cells through penetration or via natural openings such as stomata or wounds [15–17]. Wheat integrates a variety of pathways to generate type I resistance. The expression profiles of pathogen genes during the *F. graminearum* infection process imply that type I resistance may be achieved through several strategies, including improving the host's ability to recognize pathogens, triggering the outburst of extracellular hydrogen peroxide, inducing stomatal closure, and increasing the thickness of the cell wall [17].

The assessment of type I resistance is usually conducted by spraying a spore suspension onto wheat spikes at the anthesis stage and then after several days, calculating the proportion of diseased spikelets out of the total number of spikelets or diseased spikes out of the total number of treated spikes. In greenhouse experiments, type I resistance can be easily distinguished from other types of resistance because of the ease of controlling factors such as moisture, soil, fertilizer, temperature, and humidity. However, in field trials where environmental factors are uncontrollable, differentiating type I resistance from other types is difficult, and wheat with only type I resistance may also exhibit susceptibility [7]. Additionally, type I resistance is moderately to well correlated with other types of resistance [18]. To date, no wheat varieties have been found to possess complete resistance to the initial infection.

**Type II** resistance is defined as the inhibition of the spread of symptoms within the spike. The resistance mechanism is twofold: preventing pathogens from spreading from the infected spikelet into the rachis and impeding their spread from the rachis to other spikelets [19]. Many intrinsic elements of the fungus facilitate its spread within one floret and from one floret to another, such as effector proteins and mycotoxins secreted by the fungus following its successful invasion of the host cell [20]. Among the mycotoxins produced during infection, deoxynivalenol (DON), a type B trichothecene, is a particularly crucial virulence factor that aids in the spread of the fungus. It was first isolated and characterized from *Fusarium* spp. infected barley grains [21]. *F. graminearum* deficient in DON biosynthesis fails to spread within spikelets but still infect and spread in inoculated florets [16, 22]. Furthermore, it induced increased hydrogen peroxide production, leading to cell death in wheat leaves, which may aid in the transition of pathogens from a biotrophic to a necrotrophic growth phase [23]. Fusaoctaxin A, a linear octapeptide secreted or diffusible during the late infection stage, also contributes to the spread of disease symptoms, perhaps via the same mode of action as DON [24, 25]. The inhibition or elimination of the functions of the aforementioned pathogenic factors to restrict the spread of *F. graminearum* constitutes a significant facet of type II resistance in wheat.

A type II resistance assessment is conducted by injecting a spore suspension into the central florets of wheat spikes and, after a period, counting the number of diseased spikelets to measure resistance on the basis of the ratio of diseased spikelets to the total number of spikelets (percentage of symptomatic spikelets, PSS). This method is well known as the “single floret inoculation method” [26]. High-resistance wheat varieties can have a PSS below 5%, whereas highly susceptible varieties can have a PSS as high as 100% [19]. Compared with other types of resistance, type II resistance is more stable, less influenced by non-genetic factors, and easier to detect, making it the most extensively studied type of resistance.

**Type III** resistance was initially defined as resistance to kernel infection but was later redefined as kernel resistance to mycotoxin accumulation. The FGSC produces a wide range of mycotoxins, including zearalenone (ZEA) and trichothecenes [3, 27]. Mycotoxins contaminate grains and the by-products and are difficult to decompose, and ingestion by humans and livestock can result in disease symptoms, including food refusal and vomiting, thereby causing food safety issues [28]. Furthermore, as mentioned above, type B trichothecene DON, which is also known as vomitoxin because of its emetic effect on animals and humans [29], is highly beneficial for the spread of *F. graminearum* within host cells. Therefore, kernel resistance to mycotoxin accumulation is an important component of the overall resistance of wheat.

Type III resistance is assessed by harvesting the entire ear of a grain after inoculation, drying it, and then testing for toxins. In this context, the type III resistance mechanisms involved overlap partially with those of type II resistance. However, as DON is water soluble and can freely translocate between different tissues in the wheat ear, there may be mechanisms within the grain's cells that prevent the migration of DON into the cells or that expel or degrade DON [30]. Therefore, the mechanisms of type III resistance are rich in connotations.

Generally, FHB-resistant varieties show a significant positive correlation between FHB severity and DON level, whereas for susceptible and moderately susceptible varieties, the results are not significant [31]. The accumulation of DON in wheat grains varies due to differences in measurement methods, timing of measurement, concentration of inoculated spores, inoculation period, and host resistance. Compared to susceptible varieties, it cannot be absolutely concluded that the DON content is lower in the grains of moderately resistant varieties. If inoculation occurs early, the infected grains may develop into normal-sized grains due to rapid filling and thus accumulate high amounts of DON after harvest.

**Type IV** resistance confers resistance to kernel infection in wheat. It was determined by analysing the proportion of Fusarium-damaged kernels (FDK) in the total harvested kernels [32, 33]. However, a low FDK is not exclusively conferred by type IV resistance; the other types of resistance mentioned above can also lead to a reduced FDK. Thus, the definition and essence of type IV resistance require further deliberation.

**Type V** resistance refers to tolerance to yield loss. It is assessed by comparing the relative reduction in yield between diseased and healthy plants of the same variety [8]. Like type IV resistance, this type of resistance is less studied, and its definition and connotation warrant further discussion.

Notably, the aforementioned five types of resistance are classified from a genetic perspective. However, molecular biological exploration of *Arabidopsis thaliana* as a model plant has summarized the plant immune system as two-tiered, well known as pattern-triggered immunity (PTI) and effector-triggered immunity (ETI) [34]. PTI is triggered by receptor proteins on the host cell membrane (pattern-recognition receptors, PRRs) that recognize conserved molecules from pathogens (such as bacterial flagellin, fungal chitin, etc.) or other immunogenic patterns, whereas ETI is initiated by the recognition of pathogen effector proteins by host intracellular receptor proteins, primarily nucleotide-binding leucine-rich repeat receptors (NLRs) [35, 36]. Moreover, unique molecular mechanisms that confer plant disease resistance, which are mediated by proteins that differ from those mentioned above, have also been uncovered [36]. These concepts drive the cloning of resistance genes in crops and thus advance crop breeding for disease resistance [37].

With respect to wheat resistance against FHB, the understanding of the interaction between wheat and *F. graminearum* has been progressing, and emerging evidence suggests that the above PTI-ETI model is involved [20]. The separate overexpression of *CERK1* from *Haynaldia villosa*, Arabidopsis, and wheat in the FHB-susceptible wheat Fielder activated the chitin signalling pathway, which consequently increased wheat resistance to FHB [38–40]. Effectors and other virulence factors are secreted by *F. graminearum* to dampen PTI, but some of them are recognized by wheat, which then triggers another layer of resistance [41–44]. However, with the successful cloning of the genes responsible for two major FHB-resistant QTLs and the elucidation of their molecular mechanisms, the major resistance mechanisms of wheat against FHB are unique.

### Disease resistance genes and the underlying mechanisms

To date, hundreds of quantitative trait loci (QTLs) have been identified in wheat and its relatives, with the candidate genes of most of them not having been cloned [45]. Additionally, many genes related to FHB resistance have been functionally characterized, and their biological functions have been reviewed recently [20, 46]. Among the QTLs, nine (*Fhb1*-*Fhb9*) have been formally named [8, 33, 47]. However, only the genes underlying *Fhb1* and *Fhb7*, each with a major effect on FHB resistance, have been positionally cloned [48–50]. We previously described their cloning process and gene functions in detail [46]. Since then, notable progress has been made in the functional study of these two genes, and here, we summarize these new developments.

#### *Fhb1*

Two genes, *PFT*, encoding a pore-forming toxin-like protein, and *HRC* (also known as *His*), encoding a histidine-rich calcium-binding protein, have been cloned as potentially responsible for *Fhb1*-mediated resistance [48, 49, 51]. However, increasing evidence supports *HRC* as the causative gene for *Fhb1* [52–58]. Using a wealth of wheat lines with or without *PFT*, the latest study revealed that *PFT* inherently fails to confer resistance to FHB, nor does it contribute to the resistance mediated by *HRC* [59]. Furthermore, a very recent study reported that *HRC*-driven liquid‒liquid phase separation was involved in FHB resistance [60].

The sequences of the deduced proteins of the *HRC*-resistant (*HRC-R*) and *HRC*-susceptible (*HRC-S*) haplotypes differ only in the first 21 amino acids at the amino (N) terminus. Both proteins form condensates in the nucleus through liquid–liquid phase separation, with HRC-R exhibiting a weaker propensity for phase separation because of the presence of two cysteines at the N-terminus. Intriguingly, DON can trigger HRC-S condensation but not HRC-R, thereby facilitating cell death in floral organs and the rachis. Furthermore, HRC-S facilitates the development of condensates with its interacting proteins, but HRC-R counteracts this process. As most HRC-interacting proteins are involved in messenger RNA (mRNA) splicing, the positive effect of HRC-S on condensate formation results in a greater degree of alternative splicing efficiency in susceptible varieties following *F. graminearum* infection, which may determine FHB susceptibility.

The above data provide a spectacular example of how a single resistance gene can induce many immune responses and provide a unique mechanistic rationale for the stronger and more durable resistance conferred by *Fhb1*. Nevertheless, many key questions about *Fhb1* remain to be answered. Is the deletion mutation in *HRC-R* a loss- or gain-of-function mutation [61]? In most studies, the predicted open reading frame (pORF) of *HRC-R* was used for analysis, presupposing that *HRC-R* would encode a protein on the basis of this pORF. However, it remains to be determined whether the mRNA (not the pORF) produced by *HRC-R* transcription is effectively translated into a protein in wheat cells. Additionally, protein translation and accumulation are tightly regulated during plant disease resistance [36]. The change in HRC-R during *F. graminearum* infection is another critical concern. *Fhb1* exhibits inconsistent resistance levels in various wheat varieties, and at times, it fails to confer resistance to FHB in wheat [7, 62]. Two genetic loci have been found to inhibit *Fhb1*-mediated FHB resistance, but the results may not be generalizable [63]. *Fhb1* was found to confer type I, II, and IV resistance in wheat. While its response to DON has been documented, questions remain about how the pathogen invasion signal is conveyed to *Fhb1* and how DON is sensed and transmitted to *Fhb1*. *HRC* was found to be involved in calcium signalling, which is significantly involved in plant defense [55, 64]. However, the details are absent. Additionally, is *Fhb1* capable of antagonizing the inhibition of protein translation caused by the binding of DON to ribosomes? Addressing these questions will pave the way for more strategic use of the gene.

#### *Fhb7*

*Fhb7* was transferred to wheat from the wheat grass diploid *Thinopyrum elongatum* (2n = 2x = 14) and the decaploid *Th. ponticum* (2n = 10x = 70), wild relatives of wheat, respectively [65]. Its candidate gene, encoding a glutathione S-transferase (GST), possesses an exceptional ability to detoxify trichothecenes, including DON [50, 66]. In plant cells, DON resides in various cellular compartments, such as the cytoplasm, vacuoles, chloroplasts, plasmalemma, endoplasmic reticulum and ribosomes, thereby leading to changes in membrane stability and the inhibition of protein synthesis [13, 67]. In more detail, the structural basis revealed that DON binds to the aminoacyl site of the large subunit of the eukaryotic ribosome and hinders the function of peptidyl transferase to impair peptide bond formation, resulting in the inhibition of protein synthesis [68, 69]. Despite the fact that the cellular location of Fhb7-GST remains unknown—whether it shares the same location as DON or is situated elsewhere—its crystal structure has revealed a unique catalytic mechanism for DON biodegradation [70].

The protein structure of Fhb7-GST is divided into two parts: the N-terminal glutathione-binding domain (G site) and the C-terminal substrate-binding domain (H site). The G site exhibited a structure similar to that of other GSTs without DON detoxification ability, whereas the H site varied. The H site in Fhb7-GST is crucial for detoxification ability, especially the flexible loop linking two *α*-helices (*α*2 and *α*4), which may be instrumental in recognizing DON. DON toxicity is critically determined by the C12/C13 epoxy group and the C3-OH group, with the C12/C13 epoxide being the most toxic due to its inertness [71]. When glutathione is used as an antidote, Fhb7-GST specifically opens the C12/C13 epoxy group to metabolize DON into a nontoxic GSH-DON adduct. Since a few residues have been found to be crucial for Fhb7-GST catalytic activity, engineering Fhb7-GST in wheat via the CRISPR/Cas technique to yield a new version with increased activity towards DON is possible.

Additionally, the transcription of *Fhb7-GST* is highly induced by DON, and the disease resistance it confers is dependent on its expression level [50, 72]. This finding suggests an unknown mechanism within wheat cells that responds to DON. The signal transduction network likely involves a transcription activator that is induced by DON and can bind to the promoter of *Fhb7-GST*, thereby increasing its transcription. Considering the widespread adaptability of *Fhb7-GST*'s disease resistance, it is reasonable to assume that this mechanism is common in wheat and its relatives. Therefore, elucidating the mechanism and, in turn, elevating the expression of *Fhb7-GST* would be highly advantageous in maximizing the detoxification capability of this gene.

The production of DON occurs in the late stage of *F. graminearum* infection of wheat, usually following the successful colonization of wheat tissues by the pathogen, with the aim of promoting further expansion of the pathogen. The strong disease resistance provided by the *Fhb7* locus seems not to rely solely on DON detoxification catalyzed by Fhb7-GST, as Fhb7-GST functions at the late stage of pathogen infection. We recently reported that the resistance conferred by the *Fhb7* locus is multi-layered. Other genes at this locus may also be involved in resistance; moreover, the locus has triggered a reorganization of native gene expression in wheat cells after pathogen infection [73]. Together, these findings constitute the molecular rationale of *Fhb7*-mediated FHB resistance.

### Susceptibility (*S*) genes

In regard to addressing the damage to wheat caused by FHB, excessive attention has focused on identifying resistance genes while neglecting the fact that wheat is inherently susceptible to *F. graminearum*. We know relatively well how *F. graminearum* infects wheat but are not clear why wheat is susceptible to *F. graminearum*. A pioneering investigation revealed that ditelosomic lines of Chinese Spring wheat with various chromosome arm deletions displayed considerable disparities in their susceptibility to FHB, which implies that the wheat genome contains not only resistance genes but also susceptibility (*S*) genes or resistance suppressors [74]. A susceptibility factor was newly mapped on the short arm of wheat chromosome 7A [75]. Moreover, the abovementioned mode of action of *HRC-S* in susceptible wheat varieties indicates that it functions as a quintessential *S* gene [60]. Knocking out *HRC-S* in some susceptible varieties via gene editing technology can result in FHB-resistant wheat [55, 56].

However, our understanding of *S* genes in wheat is limited [76]. Given the scarcity of available resistance genes, it is worth considering the identification and modification of *S* genes that play crucial roles in the wheat-*F. graminearum* interaction as an alternative approach to managing the disease.

## Breeding strategies

**Fig. 1:**
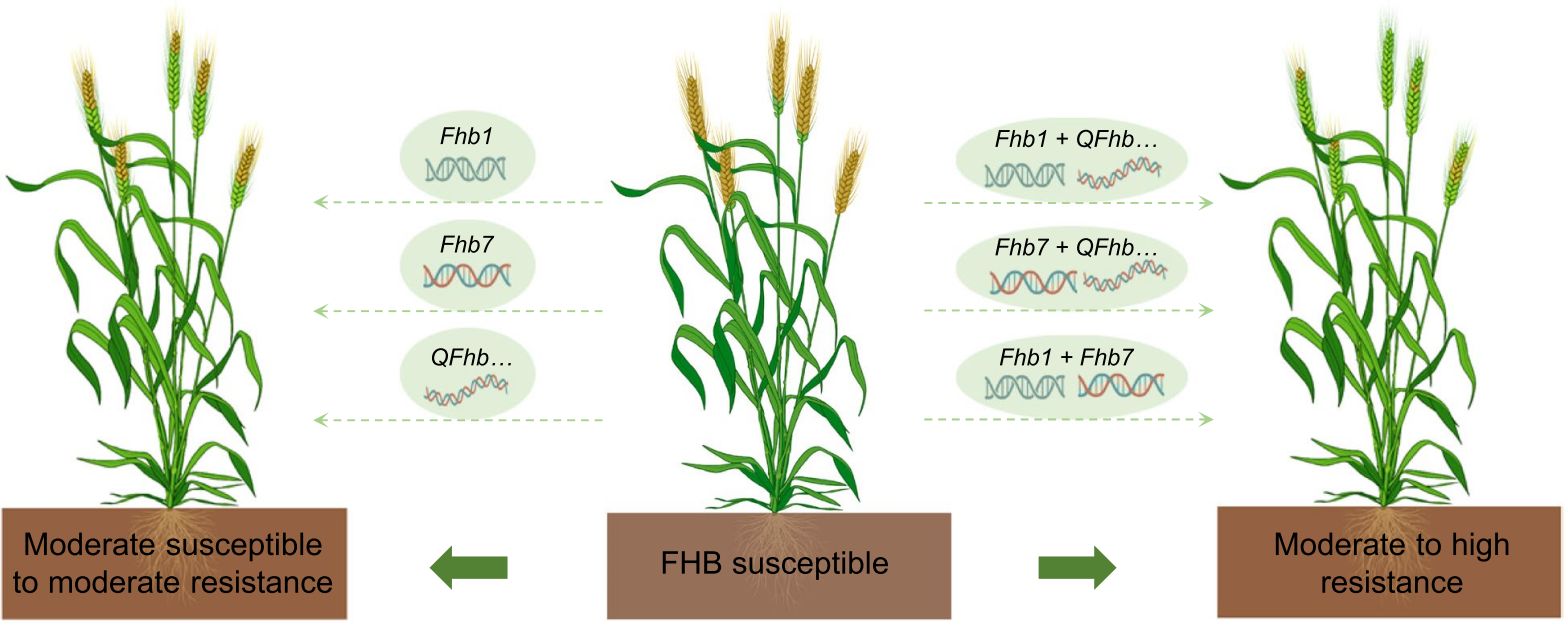
Breeding strategies using resistance genes for wheat resistance to FHB. Different disease-resistant genes or their combinations should be selected for wheat breeding according to the different resistance levels required. Utilization of *Fhb1* or *Fhb7* alone, or pyramiding minor resistance genes (*QFhb…*), can enhance the resistance of susceptible wheat varieties to moderately susceptible or moderately resistant levels. Pyramiding *Fhb1* or *Fhb7* with minor resistance genes, or combining *Fhb1* with *Fhb7*, has the potential to confer wheat with moderate to high FHB resistance. The brown spikelets in the spikes are Fusarium-damaged. The figure was created in BioRender (https://www.biorender.com/v45c873)

Despite relatively little understanding of the resistance mechanisms against wheat FHB, considerable progress has been made in breeding for FHB resistance in wheat, particularly in the middle and lower reaches of the Yangtze River in China, where wheat varieties exhibit the best resistance to the disease [9]. The exploitation of resistance genes in wheat breeding for FHB resistance can be summarized into three principal strategies, each employing different resistance genes and resulting in varying levels of resistance (Fig. 1).

### Utilization of *Fhb1* or *Fhb7* alone or pyramiding minor resistance genes

The deployment of *Fhb1* has been a significant strategy in wheat breeding worldwide for enhancing FHB resistance [77–79]. This major QTL has a substantial and stable effect on resistance, yielding wheat varieties with resistance levels ranging from moderately susceptible to moderately resistant [80]. *Fhb1* in global wheat varieties can be traced back to at least two donors, Sumai3 and Ningmai9. Sumai3 is popular worldwide, whereas Ningmai9 is the main donor of Chinese wheat varieties [77, 78].

**Fig. 2:**
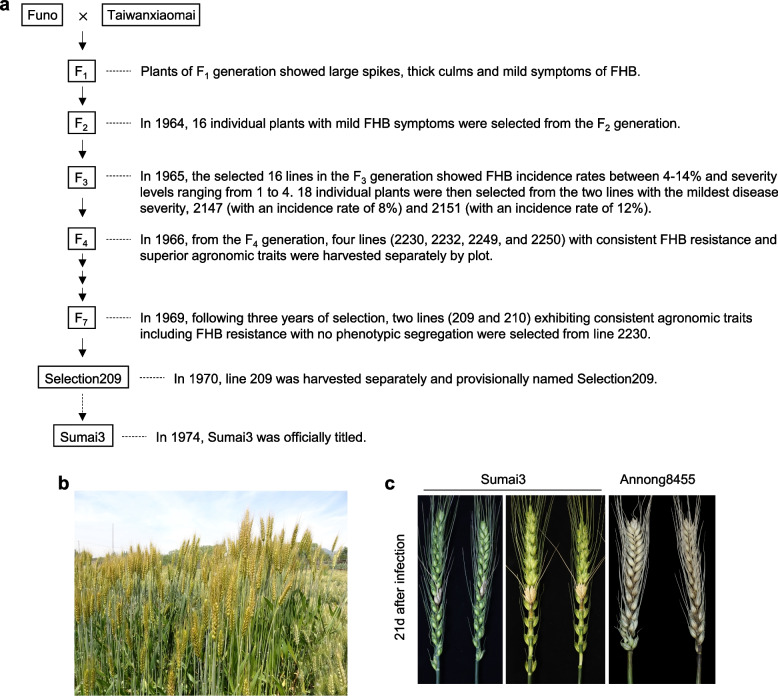
Sumai3 harboring the *Fhb1* locus is highly resistant to Fusarium head blight, yet its agronomic traits are less desirable. **a** Details of the breeding process for Sumai3. The diagram was generated based on the descriptions provided in the published article (Liu et al. 1988). **b** Phenotypes of Sumai3 in the field. Note its tall plant height. **c** Disease symptoms after infection with *F. graminearum* strain PH-1 using single floret inoculation method. Wheat variety Annong8455 serves as a susceptible control

Sumai3, developed in 1970 and officially named in 1974, originated from the cross of Funo and Taiwanxiaomai by the Suzhou Regional Institute of Agricultural Sciences in Jiangsu Province, China [81] (Fig. 2). *Fhb1* in Sumai3 was derived from Taiwanxiaomai, a Chinese landrace with moderate FHB resistance [82]. Although Sumai3 is highly resistant to FHB and has a low DON content in infected grains, it is not used as a commercial variety but rather as a valuable parent in breeding programs because of its undesirable agronomic traits, such as tall plant height and poor yield potential [31] (Fig. 2).

Ningmai9 was developed from a cross of Yangmai6 and Norin129 by the Jiangsu Academy of Agricultural Sciences, China, and was released in 1997 [9]. It is moderately resistant to FHB. The *Fhb1* locus in Ningmai9 is traceable to the Japanese variety Norin129, which is itself derived from the Japanese landrace Shinchunaga [77]. Notably, genetic relationship analysis revealed that Shinchunaga is closer to Chinese wheat varieties than to Japanese landraces and that resistance QTLs, including *Fhb1* in Shinchunaga, may originate from Chinese landraces [80, 82]. In addition to moderate resistance to FHB, Ningmai9 has desirable agronomic traits with a high number of spikes and grains per spike. Thirty varieties derived from Nanmai9 with moderate resistance to FHB were released for wheat production [9].

The application of the *Fhb7* locus in breeding efforts has been notably effective. Three *Fhb7* alleles, two (*Fhb7*^*The1*^ and *Fhb7*^*The2*^) from diploid *Th. elongatum* and one (*Fhb7*^*Thp*^) from the decaploid *Th. ponticum*, have been integrated into wheat genomes and subsequently used in wheat breeding [83–86].

*Fhb7*^*Thp*^ was introduced into the high-yielding wheat variety Jimai22 by Dr. Kong and colleagues, who successfully cloned *Fhb7-GST*. The subsequently developed wheat variety, Shannong48, showed moderate resistance to FHB and was released in 2021. Since *Fhb7*^*Thp*^ is closely linked to the *PSY-E2* gene encoding phytoene synthase, the grains of Shannong48 are also rich in carotenoids [87]. However, the close linkage between the two genes has been successfully broken in breeding through molecular marker-assisted selection techniques [87]. *Fhb7*^*The*^ was also introduced into the wheat variety Jimai22 by Dr. Han’s laboratory [86]. The developed wheat varieties Zhongke166 and Zhongke1878 showed moderate resistance to FHB and were released in 2022 and 2024, respectively [86].

Stacking minor resistance genes is successful in breeding FHB-resistant wheat. A notable case is Yangmai158, a very popular variety in the middle and lower reaches of the Yangtze River in China [9]. Despite the absence of the *Fhb1* and *Fhb7* loci, Yangmai158 possesses multiple minor loci that collectively confer a moderate level of resistance to FHB [88–90]. Recently, our laboratory has conducted fine mapping of some of these loci and successfully cloned the corresponding genes (unpublished results). These findings provide a significant foundation for advancing FHB management.

### Pyramiding *Fhb1* or *Fhb7* with minor resistance genes for enhanced resistance

In some breeding programs, *Fhb1* or *Fhb7* is strategically stacked with other minor resistance genes to create a pyramiding effect. This pyramiding approach has proven to be more efficient and stable in increasing FHB resistance levels than the use of a single gene.

Yangmai33 (Sumai6/97G59//Yangmai18; referred to as Yang16-157 during the variety trial), which integrates *Fhb1* into the Yangmai lineage and was released in 2021, presents FHB resistance comparable to that of Sumai 3 [91]. The donor of *Fhb1* was Ningmai9, which is a recurrent parent of Yangmai18 (Ningmai9^4^///Yangmai158^6^//88–128/NNP045). In addition to its high resistance to FHB, Yangmai33 retains superior agricultural characteristics, such as high productivity [91]. Recently, our laboratory introduced *Fhb7*^*The*^ into Yangmai158 and yielded a novel germplasm, Yangnongmai158, with FHB resistance comparable to that of Sumai 3 [73]. These findings indicate that the combination of *Fhb1* (or *Fhb7*) with minor resistance genes from Yangmai varieties can further increase resistance to FHB. These findings further suggest that the resistance mechanisms mediated by *Fhb1* (or *Fhb7*) and these minor genes are distinct but synergistic.

### Combination of *Fhb1* and *Fhb7*

As the two most potent resistance loci, *Fhb1* and *Fhb7* mediate distinct resistance mechanisms. This implies that their combination can markedly increase the level of wheat resistance to FHB. *Fhb7*^*The*^ has been introduced into wheat varieties harboring *Fhb1*, resulting in wheat lines with higher FHB resistance than their parents harboring *Fhb7*^*The*^ or *Fhb1* alone [92]. The combination of *Fhb7*^*Thp*^ and *Fhb1* has also been shown to be more effective in improving wheat resistance to FHB than the use of a single gene [93]. In our laboratory, we crossed Yangnongmai158, which carries *Fhb7*^*The*^, with wheat varieties harboring *Fhb1*, yielding novel germplasms with the combination of *Fhb7*^*The*^ and *Fhb1*, resulting in high FHB resistance comparable to that in Sumai3 (unpublished results). Although highly resistant FHB varieties containing both *Fhb1* and *Fhb7* have not yet been successfully developed, the strategy of combining these two genes for increased resistance has been shown to be effective.

## Conclusions and challenges

Although the classification and definition of FHB resistance in wheat have long been proposed, the molecular mechanisms underlying this resistance remain obscure. The molecular basis of quantitative disease resistance in plants varies and is difficult to define [94, 95]. The functions of the cloned FHB resistance genes in wheat or its relatives are distinct from those of other resistance genes generally accepted in plants. Additionally, the regulation of the cloned genes remains vague. A full understanding of the resistance mechanisms of wheat against FHB remains an ongoing pursuit.

However, through decades of persistent efforts, significant progress has been made in controlling FHB through wheat breeding programs, facilitating the continuous release of wheat varieties with improved FHB resistance. The successful breeding of these varieties indicates that the use of *Fhb1* or *Fhb7* alone or in combination with other minor resistance genes represents a layered resistance strategy in wheat breeding. Therefore, cloning these unknown minor-effect resistance genes and elucidating their mechanisms are highly important. However, owing to the minor resistance effect of each gene, successful cloning of these genes requires well-designed strategies and technological breakthroughs. For example, high-quality chromosome segment substitution line populations can facilitate the successful cloning of these minor-effect QTLs. Additionally, advances and cost reductions in whole-genome sequencing have made it easier to obtain the whole genome of one wheat variety. Together with the development of gene editing technologies, cloning these minor QTLs will become more feasible [96, 97]. For *Fhb1* and *Fhb7*, in-depth analysis of their regulation will provide insights for maximizing the utilization of these resistance genes.

Furthermore, the identification and functional characterization of FHB susceptibility genes in wheat are highly important. The details of *F. graminearum*-wheat interaction are also critical for comprehensively understanding wheat resistance to this disease. Identifying the target genes of fungal virulence factors (e.g., DON, effector proteins) in wheat, along with their regulatory networks, and exploring natural variations in these target genes or key components of their networks in wheat and its relatives will provide valuable genetic resources for breeding.

As there are different types of resistance to FHB in wheat, a strategy could be designed to establish a multifaceted defense against FHB, wherein different resistance genes operate within distinct defense pathways, collectively enhancing protection against FHB. Primarily, breeding programs must strike a balance between disease resistance and various other traits, including yield, rather than focusing solely on disease resistance improvement [98]. FHB resistance and yield in wheat are usually antagonistic; however, the molecular mechanisms underlying this trade-off remain elusive. However, numerous wheat varieties with both increased FHB resistance and improved yield have been released. Cloning the resistance genes in these varieties and analysing their regulatory networks will clarify the molecular basis of the trade-off between FHB resistance and yield or other agronomic traits, thereby facilitating the development of more wheat varieties with both high FHB resistance and desirable agronomic traits. However, on the basis of current breeding experience, enhancing the FHB resistance of wheat varieties to an immune level remains a formidable challenge. Given the capricious nature of climate change and the shifts in the population structure of the FGSC, breeding for FHB resistance is an ongoing challenge.

## Data Availability

Not applicable.
